# Supplementary material for: COZOID: contact zone identifier for visual analysis of protein-protein interactions
Source: BMC Bioinformatics. 2018 Apr 6;19:125. doi: 10.1186/s12859-018-2113-6 (PMC5889581; doi:10.1186/s12859-018-2113-6)
Supplement: Supplementary file 2 — Software build. Executable binary file of the software tool. (ZIP 118,784 kb) [file 12859_2018_2113_MOESM2_ESM.zip › cozoid/java/docs/beansbinding-1.2.1-doc.zip!/index-files/index-22.html]

V-Index (Beans Binding)


---


|  |  |  |
| --- | --- | --- |
| **Overview** \| Package \| Class \| Use \| **Tree** \| **Deprecated** \| **Index** \| **Help** | |  |
| **PREV LETTER**   **NEXT LETTER** | **FRAMES**    **NO FRAMES**     **All Classes** |  |


A B C D E F G H I J K L M N O P Q R S T U V W 

---


## **V**

**validate(T)** - Method in class org.jdesktop.beansbinding.Validator: Validates a value; returns `null` for a valid value, and a `Result` object describing the problem for an invalid value. **Validator**<T> - Class in org.jdesktop.beansbinding: `Validator` is responsible for validating the value from the target of a `Binding`. **Validator()** - Constructor for class org.jdesktop.beansbinding.Validator: **Validator.Result** - Class in org.jdesktop.beansbinding: An instance of `Result` is returned from a `Validator's` `validate` method to indicate an invalid value. **Validator.Result(Object, String)** - Constructor for class org.jdesktop.beansbinding.Validator.Result: Creates a `Result` with the given error code and description. **Value()** - Method in class org.jdesktop.el.impl.parser.ELParser: **valueAt(int, int)** - Method in class org.jdesktop.swingbinding.impl.ListBindingManager: **valueChanged(int, int)** - Method in class org.jdesktop.swingbinding.impl.ListBindingManager: **ValueExpression** - Class in org.jdesktop.el: An `Expression` that can get or set a value. **ValueExpression()** - Constructor for class org.jdesktop.el.ValueExpression: **ValueExpressionImpl** - Class in org.jdesktop.el.impl: An `Expression` that can get or set a value. **ValueExpressionImpl()** - Constructor for class org.jdesktop.el.impl.ValueExpressionImpl: **ValueExpressionImpl(String, Node, FunctionMapper, VariableMapper, Class)** - Constructor for class org.jdesktop.el.impl.ValueExpressionImpl: **ValueExpressionLiteral** - Class in org.jdesktop.el.impl: **ValueExpressionLiteral()** - Constructor for class org.jdesktop.el.impl.ValueExpressionLiteral: **ValueExpressionLiteral(Object, Class)** - Constructor for class org.jdesktop.el.impl.ValueExpressionLiteral: **valueOf(String)** - Static method in enum org.jdesktop.beansbinding.AutoBinding.UpdateStrategy: Returns the enum constant of this type with the specified name. **valueOf(String)** - Static method in enum org.jdesktop.beansbinding.Binding.SyncFailureType: Returns the enum constant of this type with the specified name. **valueOf(String)** - Static method in enum org.jdesktop.el.Expression.Result.Type: Returns the enum constant of this type with the specified name. **ValuePrefix()** - Method in class org.jdesktop.el.impl.parser.ELParser: **values()** - Static method in enum org.jdesktop.beansbinding.AutoBinding.UpdateStrategy: Returns an array containing the constants of this enum type, in the order they are declared. **values()** - Static method in enum org.jdesktop.beansbinding.Binding.SyncFailureType: Returns an array containing the constants of this enum type, in the order they are declared. **values()** - Static method in enum org.jdesktop.el.Expression.Result.Type: Returns an array containing the constants of this enum type, in the order they are declared. **ValueSuffix()** - Method in class org.jdesktop.el.impl.parser.ELParser: **VariableMapper** - Class in org.jdesktop.el: The interface to a map between EL variables and the EL expressions they are associated with. **VariableMapper()** - Constructor for class org.jdesktop.el.VariableMapper: **VariableMapperFactory** - Class in org.jdesktop.el.impl.lang: **VariableMapperFactory(VariableMapper)** - Constructor for class org.jdesktop.el.impl.lang.VariableMapperFactory: **VariableMapperImpl** - Class in org.jdesktop.el.impl.lang: **VariableMapperImpl()** - Constructor for class org.jdesktop.el.impl.lang.VariableMapperImpl: **visit(Node)** - Method in class org.jdesktop.el.impl.lang.ExpressionBuilder: **visit(Node)** - Method in interface org.jdesktop.el.impl.parser.NodeVisitor

---


|  |  |  |
| --- | --- | --- |
| **Overview** \| Package \| Class \| Use \| **Tree** \| **Deprecated** \| **Index** \| **Help** | |  |
| **PREV LETTER**   **NEXT LETTER** | **FRAMES**    **NO FRAMES**     **All Classes** |  |


A B C D E F G H I J K L M N O P Q R S T U V W 

---
